# Intramitochondrial proteostasis is directly coupled to α-synuclein and amyloid β1-42 pathologies

**DOI:** 10.1074/jbc.RA119.011650

**Published:** 2020-05-08

**Authors:** Janin Lautenschläger, Sara Wagner-Valladolid, Amberley D. Stephens, Ana Fernández-Villegas, Colin Hockings, Ajay Mishra, James D. Manton, Marcus J. Fantham, Meng Lu, Eric J. Rees, Clemens F. Kaminski, Gabriele S. Kaminski Schierle

**Affiliations:** 1 Molecular Neuroscience Group, Department of Chemical Engineering and Biotechnology, University of Cambridge, West Cambridge Site, Philippa Fawcett Drive, Cambridge, United Kingdom; 2 Quantitative Imaging Group, Department of Chemical Engineering and Biotechnology, University of Cambridge, West Cambridge Site, Philippa Fawcett Drive, Cambridge, United Kingdom; 3 Laser Analytics Group, Department of Chemical Engineering and Biotechnology, University of Cambridge, West Cambridge Site, Philippa Fawcett Drive, Cambridge, United Kingdom

**Keywords:** α-synuclein, amyloid-β (Aβ,), mitochondria, neurodegeneration, HtrA2/Omi, HtrA serine peptidase 2, Parkinson disease, protein aggregation, protein homeostasis, Lon protease, Lon peptidase 1 mitochondrial, α-synuclein (a-synuclein), amyloid-β (AB), mitochondria, neurodegenerative disease, neurodegeneration, HtrA serine peptidase 2, HtrA2/Omi, protein homeostasis

## Abstract

Mitochondrial dysfunction has long been implicated in the neurodegenerative disorder Parkinson's disease (PD); however, it is unclear how mitochondrial impairment and α-synuclein pathology are coupled. Using specific mitochondrial inhibitors, EM analysis, and biochemical assays, we report here that intramitochondrial protein homeostasis plays a major role in α-synuclein aggregation. We found that interference with intramitochondrial proteases, such as HtrA2 and Lon protease, and mitochondrial protein import significantly aggravates α-synuclein seeding. In contrast, direct inhibition of mitochondrial complex I, an increase in intracellular calcium concentration, or formation of reactive oxygen species, all of which have been associated with mitochondrial stress, did not affect α-synuclein pathology. We further demonstrate that similar mechanisms are involved in amyloid-β 1-42 (Aβ42) aggregation. Our results suggest that, in addition to other protein quality control pathways, such as the ubiquitin–proteasome system, mitochondria *per se* can influence protein homeostasis of cytosolic aggregation-prone proteins. We propose that approaches that seek to maintain mitochondrial fitness, rather than target downstream mitochondrial dysfunction, may aid in the search for therapeutic strategies to manage PD and related neuropathologies.

Parkinson's disease (PD) is the second most common neurodegenerative disease and affects about 1% of the population over 60 years (1). α-Synuclein aggregation has been found central to the disease, because SNCA mutations are associated with familiar PD (2) and α-synuclein has been identified as a major constituent of Lewy bodies in sporadic PD and dementia with Lewy bodies (3). The relationship between protein aggregation and protein levels is well established in cases of familiar PD involving SNCA gene duplication and triplication (4–6), however, what triggers protein aggregation in sporadic cases of PD is less clear. Likewise, Alzheimer's disease is linked to increased protein aggregation, where enhanced levels of amyloid-β (Aβ) are found due to mutations in the genes coding for the amyloid precursor protein (7, 8) or presenilin (9–11).

Mitochondria have long been implicated in PD, ever since the discovery that inhibitors of the mitochondrial complex I can lead to dopaminergic neuron death (12–16). Furthermore, the regulation of mitophagy via the PTEN-induced kinase 1 (PINK1) plays a role in PD (17) and seems to be coupled to α-synuclein toxicity. PINK1 overexpression is able to decrease the effect of α-synuclein toxicity in *Drosophila* (18, 19), and PINK1 knockout in mice increases α-synuclein neurotoxicity (20, 21). Furthermore, PINK1 iPSC-derived midbrain dopaminergic neurons show accumulation and aggregation of α-synuclein (22), and PINK1 knockout rats display α-synuclein *de novo* aggregation (23).

We have demonstrated previously that α-synuclein interaction with calcium leads to conformational changes at the C terminus of α-synuclein, but also at the aggregation-prone nonamyloid component (NAC) region, suggesting that calcium can directly influence the aggregation propensity of α-synuclein (24). Thus, we tested whether treatment with BAPTA-AM, which is supposed to decrease intracellular calcium by calcium chelation, was able to decrease α-synuclein pathology. Surprisingly, prolonged incubation with BAPTA-AM significantly enhanced α-synuclein aggregation. We could show that BAPTA-AM treatment was accompanied by mitochondrial fragmentation, which led us to study and show that disturbances in intra-mitochondrial proteostasis could aggravate α-synuclein aggregation. We identified that the Lon protease and the high-temperature requirement protein A2 (HtrA2) protease, as well as mitochondrial protein import were crucial in determining the level of α-synuclein aggregation. However, inhibition of the mitochondrial complex I and a direct increase in cytosolic calcium or oxidative stress were not able to increase α-synuclein aggregation after seeding such as observed upon inhibition of mitochondrial protein homeostasis. In addition, inhibition of the mitochondrial protease HtrA2 and blocking mitochondrial protein import also increased Aβ42 aggregation and we could show that isolated mitochondria were directly capable to diminish Aβ42 aggregation *in vitro*.

## Results

### Prolonged BAPTA-AM treatment of cells increases α-synuclein pathology

SH-SY5Y cells overexpressing YFP–α-synuclein were incubated for 4 h with small fibrillar seeds made of unlabeled human recombinant α-synuclein to study α-synuclein pathology after seeding, as described previously (25–29). Cells were left in culture for 3 days before the level of α-synuclein aggregation within the cells was determined (see Fig. S1, *A* and *B*, for treatment regime and fibrillar seeds). Although unseeded YFP–α-synuclein overexpressing cells did not display any aggregates, those that were seeded displayed large YFP–α-synuclein-positive aggregates, which were built up from fine filaments (Fig. S1*C*). Furthermore, YFP–α-synuclein inclusions stained positive for ubiquitin and p62, which are both characteristic markers of Lewy bodies in human disease (25) (Fig. S1, *D* and *E*).

**Figure 1.**
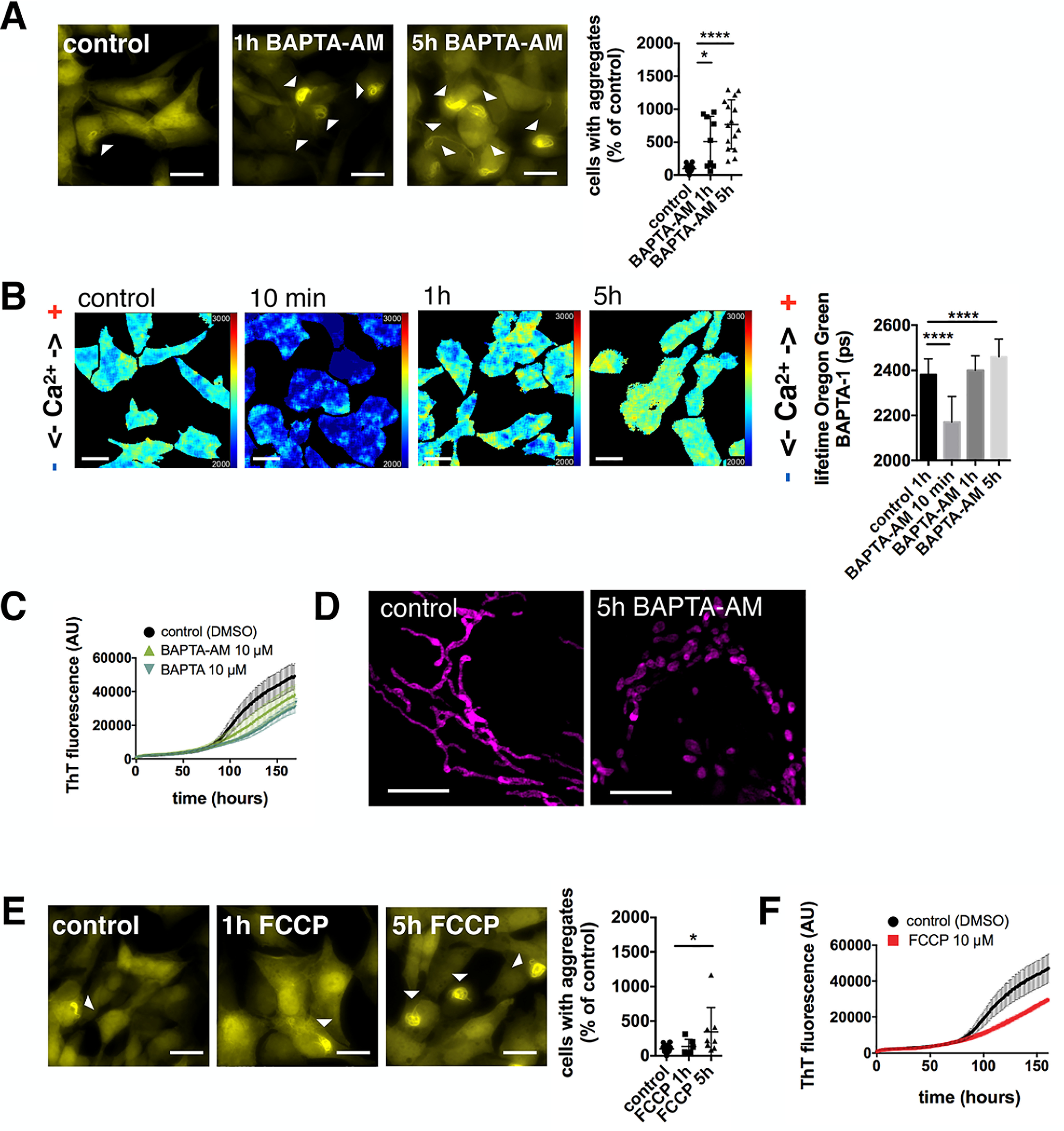
**BAPTA-AM treatment increases α-synuclein pathology.** *A,* YFP–α-synuclein SH-SY5Y cells were treated with DMSO (control), 10 μm BAPTA-AM for 1 h (before fibrillar seed incubation) and for 5 h (before plus during the incubation with α-synuclein fibrillar seeds). *Scale bars*: 20 μm. α-Synuclein seeding was increased upon 1 h pre-treatment and 5 h treatment with BAPTA-AM. Data are presented as mean ± S.D. *, *p* = 0.0127 and ****, *p* < 0.0001 (Kruskal-Wallis test with Dunn's multiple comparison). *n* = 16, 9, 15 with *n* = regions analyzed, three biological repeats. *B,* fluorescence lifetime images of cytosolic calcium levels (Oregon Green^TM^ 488 BAPTA-1 fluorescence lifetime) in SH-SY5Y cells treated with DMSO (control), 10 μm BAPTA-AM for 10 min, 1 or 5 h. *Scale bars*: 20 μm. The cytosolic calcium level within cells was significantly reduced upon 10 min incubation with BAPTA-AM, however, after 1 h of incubation with BAPTA-AM calcium levels returned back to basal levels. After 5 h treatment with BAPTA-AM, calcium levels significantly increased beyond basal calcium levels. Data are presented as mean ± S.D. ****, *p* < 0.0001 (Kruskal-Wallis test with Dunn's multiple comparison). *n* = 88, 54, 61, and 46, with *n* = cells analyzed, three biological repeats. *C,* ThT assay displaying the aggregation kinetics of α-synuclein *in vitro* in the presence of DMSO, 10 μm BAPTA-AM, or 10 μm BAPTA. Data are presented from three biological repeats. *D,* mito-RFP stained mitochondrial network in SH-SH5Y cells. Cells were treated with DMSO (control) or 10 μm BAPTA-AM for 5 h. *Scale bars*: 5 μm. *E,* YFP–α-synuclein overexpressing SH-SY5Y cells treated with DMSO (control), 10 μm FCCP for 1 h (before fibrillar seed incubation) and 5 h (before plus during the incubation with α-synuclein fibrillar seeds). *Scale bars*: 20 μm. α-Synuclein aggregation was increased upon 5 h treatment with FCCP. Data are presented as mean ± S.D. *, *p* = 0.0374 (Kruskal-Wallis test with Dunn's multiple comparison). *n* = 9, 6, and 8 with *n* = regions analyzed, three biological repeats. *F,* ThT assay displaying the aggregation kinetics of α-synuclein *in vitro* in the presence of DMSO or 10 μm FCCP. Data are presented from three biological repeats.

We have previously shown that α-synuclein interacts strongly with calcium, leading to conformational changes both at the C-terminal calcium-binding domain, and the aggregation-prone NAC region, which suggests that calcium can directly influence the aggregation propensity of α-synuclein. Consistently, increased calcium concentrations significantly enhanced α-synuclein nucleation *in vitro* (24). BAPTA-AM, a calcium chelator, is supposed to decrease cytosolic calcium and has previously been reported to alleviate KCl-induced α-synuclein aggregation (30). However, when we treated the above described cells with BAPTA-AM before the incubation with fibrillary seeds (1 h) or before and during incubation with fibrillary seeds (5 h), α-synuclein aggregation was drastically increased (Fig. 1*A*). We thus tested the effect of BAPTA-AM in SH-SY5Y cells and verified that BAPTA-AM was able to decrease cytosolic calcium. However, the calcium buffering achieved by BAPTA-AM was only transient and cytosolic calcium concentrations were already back to control levels after longer treatment with BAPTA-AM, which is due to the cells compensating for reduced calcium levels (Fig. 1*B*, fluorescence lifetime decrease after 10 min from 2381 ± 8 ps to 2170 ± 15 ps, *p* < 0.0001, lifetime of 2400 ± 8 ps after 1 h and 2460 ± 12 ps after 5 h). Because the 1 h treatment of cells with BAPTA-AM led to calcium levels comparable with control but already to increased α-synuclein aggregation suggested that the increase of α-synuclein aggregation by BAPTA-AM was not directly mediated by increased intracellular calcium concentrations. In addition, we tested whether both the ester form of BAPTA, BAPTA-AM, as well as the active BAPTA itself were directly capable to affect the aggregation of α-synuclein. We found no difference in α-synuclein aggregation kinetics measured *in vitro* by thioflavin T (ThT) fluorescence in the presence of BAPTA and BAPTA-AM (Fig. 1*C*, *t*_50_ 125.6 ± 8.6 h and 122.6 ± 7.2 h *versus* 116.6 ± 11.1 h) confirming that the effect of BAPTA is most likely triggered by a cellular response. We consequently discovered a previous publication showing that BAPTA-AM could lead to mitochondrial fragmentation (31). We thus stained the cells with mitochondria-RFP, a mitochondrial marker, and showed that prolonged BAPTA-AM treatment of cells led to mitochondrial fragmentation (Fig. 1*D*).

Thus, we hypothesized that mitochondrial dysfunction may influence α-synuclein aggregation *per se*, which we tested by treating cells with carbonyl cyanide 4-(trifluoromethoxy)phenylhydrazone (FCCP), a mitochondrial uncoupler that dissipates the mitochondrial membrane potential. Treatment of cells with FCCP during α-synuclein fibril incubation (5 h) significantly increased α-synuclein aggregation (Fig. 1*E*). To test whether FCCP did not increase α-synuclein aggregation *per se*, we also performed an *in vitro* aggregation assay and showed that FCCP was not capable of influencing α-synuclein aggregation directly (*t*_50_ 117.0 ± 9.8 h *versus* 115.6 ± 10.1 h). We thus confirmed that the effect of FCCP treatment in cells is the result of a cellular response rather than of a direct interaction of FCCP with α-synuclein (Fig. 1*F*).

### Classical downstream effectors of mitochondrial dysfunction are unable to influence α-synuclein pathology

**Figure 2.**
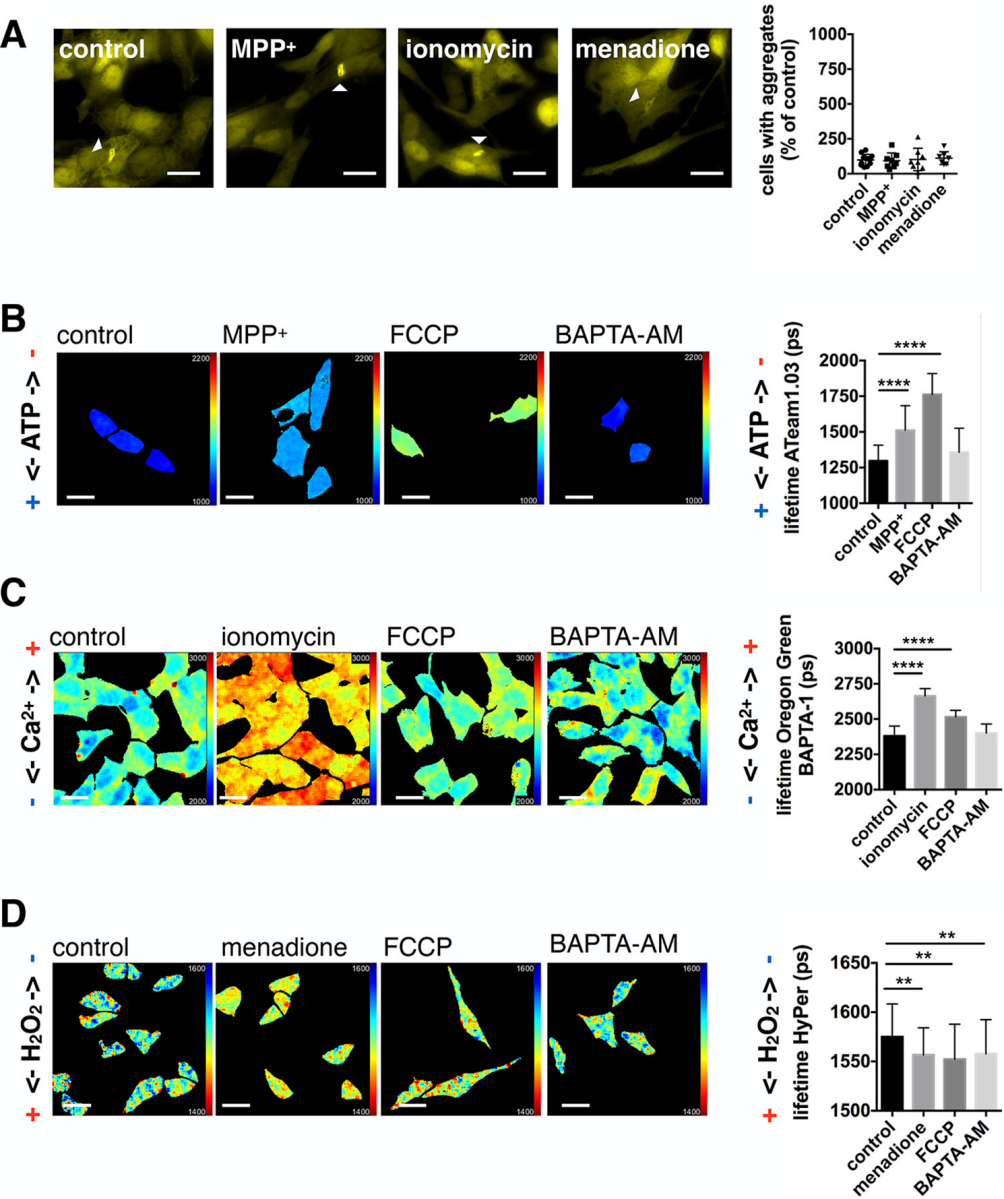
**Downstream effectors of mitochondrial dysfunction do not influence α-synuclein pathology.** *A,* YFP–α-synuclein SH-SY5Y cells were treated with DMSO (control), 500 μm MPP^+^, 1 μm ionomycin, or 3 μm menadione for 3 days (1 h before, during α-synuclein fibrillar seed incubation, and during the 3-day period until evaluation). *Scale bars*: 20 μm. α-Synuclein seeding was not significantly increased (one-way ANOVA with Dunnett's post hoc correction). Data are presented as mean ± S.D., *n* = 11, 8, 8, and 7 with *n* = regions analyzed, three biological repeats. *B,* fluorescence lifetime images and graphs for ATP levels (Ateam1.03 donor fluorescence lifetime) in SH-SY5Y cells treated with DMSO (control), 500 μm MPP^+^, 10 μm FCCP, and 10 μm BAPTA-AM for 1 h. MPP^+^ and FCCP significantly decreased ATP levels, BAPTA-AM had no significant effect. ****, *p* < 0.0001 and *n* = 43, 74, 48, and 47. *C,* fluorescence lifetime images and graphs for cytosolic calcium levels (Oregon Green^TM^ 488 BAPTA-1 fluorescence lifetime) in SH-SY5Y cells treated with DMSO (control), 1 μm ionomycin, 10 μm FCCP, and 10 μm BAPTA-AM for 1 h. Ionomycin and FCCP significantly increased cytosolic calcium levels. ****, *p* < 0.0001 and *n* = 88, 60, 42, and 61. *D,* fluorescence lifetime images and graphs of H_2_O_2_ levels (HyPer fluorescence lifetime) in SH-SY5Y cells treated with DMSO (control), 3 μm menadione, 10 μm FCCP, and 10 μm BAPTA-AM for 1 h. Menadione, FCCP, and BAPTA-AM significantly increased H_2_O_2_ levels. Both, FCCP and BAPTA-AM did not produce higher H_2_O_2_ levels than seen with menadione. **, *p* = 0.0017, *p* = 0.0012, and *p* = 0.0058 and *n* = 79, 63, 36, and 70. All *scale bars*: 20 μm. All data are presented as mean ± S.D. with *n* = cells analyzed, Kruskal-Wallis test with Dunn's multiple comparison, three biological repeats.

We next tested whether downstream events of mitochondrial dysfunction could reproduce increased α-synuclein aggregation. We therefore used 1-methyl-4-phenylpyridinium (MPP^+^), the active metabolite of 1-methyl-4-phenyl-1,2,3,6-tetrahydropyridine to inhibit complex I of the electron transport chain, which inhibits mitochondrial ATP production. We used ionomycin, an ionophore, to directly increase cytosolic calcium concentrations via calcium influx through the plasma membrane, and we used menadione to induce the formation of reactive oxygen species via redox cycling (32). However, when YFP–α-synuclein overexpressing SH-SY5Y cells with α-synuclein seeds were treated for 3 days, no increase in α-synuclein aggregation could be detected (Fig. 2*A*). To test that the various inhibitors were active, we measured ATP, calcium, and H_2_O_2_ levels in SH-SY5Y cells using the fluorescent sensors Ateam1.03 (33, 34), Oregon-Green^TM^ BAPTA-1, and HyPer (35), respectively. The readout of the fluorescence lifetime of these sensors permits to estimate and directly compare the effect of our different treatments (36, 37). Our results show that MPP^+^-induced inhibition of complex I reduced ATP levels (Fig. 2*B*, fluorescence lifetime increase of the FRET donor from 1298 ± 17 to 1511 ± 20 ps, *p* < 0.0001), ionomycin treatment of cells increased cytosolic calcium concentrations (Fig. 2*C*, fluorescence lifetime increase of Oregon-Green^TM^ BAPTA-1 from 2381 ± 8 to 2663 ± 7 ps, *p* < 0.0001), and menadione treatment increased H_2_O_2_ levels in cells (Fig. 2*D*, fluorescence lifetime decrease of cpYFP from 1575 ± 4 to 1557 ± 3 ps, *p* = 0.0017). Treatment of cells with MPP^+^ lead to less ATP depletion than treatment of cells with FCCP, but to higher ATP depletion than following BAPTA-AM treatment (Fig. 2*B*). Ionomycin treatment led to a higher calcium increase compared with both FCCP and BAPTA-AM treatments (Fig. 2*C*). Moreover, menadione treatment of cells led to a comparable increase in H_2_O_2_ concentrations than treatment with FCCP and BAPTA-AM (Fig. 2*D*). In summary, the above experiments show that there is no correlation between a loss in ATP-levels, an increase in calcium or H_2_O_2_ concentrations, and increased α-synuclein pathology.

### Inhibition of mitochondrial proteostasis increases α-synuclein pathology

**Figure 3.**
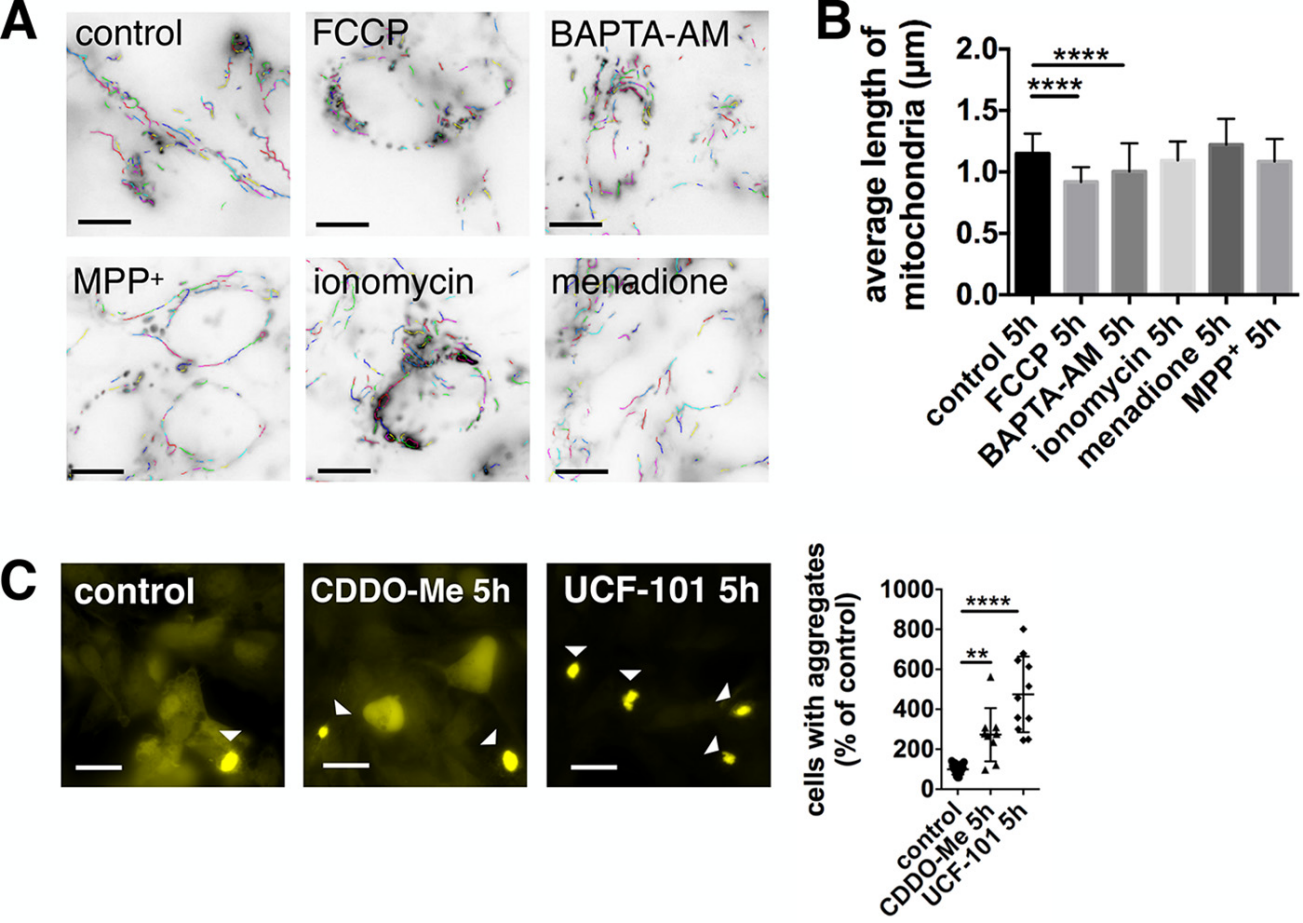
**Inhibition of mitochondrial proteases increases α-synuclein pathology.** *A* and *B,* quantification of mitochondrial fragmentation after 5 h treatment with 10 μm FCCP, 10 μm BAPTA-AM, 500 μm MPP^+^, 1 μm ionomycin, and 3 μm menadione. The mitochondrial length was significantly decreased after treatment with FCCP and BAPTA-AM. *Scale bars*: 10 μm. Data are presented as mean ± S.D. ****, *p* < 0.0001 (Kruskal-Wallis test with Dunn's multiple comparison). *n* = 76, 92, 103, 90, 88, and 89 with *n* = individual images, three biological repeats. Image analysis of mitochondrial fragmentation was performed using NIEL Mito (91). *C,* YFP–α-synuclein SH-SY5Y cells were treated with DMSO (control), 1 μm CDDO-Me, or 20 μm UCF-101 before and during the incubation with α-synuclein fibrillar seeds (*5h*). *Scale bars*: 20 μm. α-Synuclein aggregation was increased upon both treatments. Data are presented as mean ± S.D. **, *p* = 0.005 and ****, *p* < 0.0001 (Kruskal-Wallis test with Dunn's multiple comparison). *n* = 15, 9, and 11 with *n* = regions-analyzed, three biological repeats.

In the next step, we evaluated the level of mitochondrial fragmentation upon treatment with FCCP and BAPTA-AM, as well as MPP^+^, ionomycin, and menadione. Automated analysis of mitochondrial length showed that both FCCP and BAPTA-AM treatments led to mitochondrial fragmentation, whereas neither MPP^+^, nor ionomycin or menadione did (Fig. 3, *A* and *B*). Furthermore, we saw that FCCP treatment led to higher levels of mitochondrial fragmentation compared with BAPTA-AM, although the effect of BAPTA-AM on α-synuclein aggregation was more pronounced than upon FCCP treatment (see to Fig. 1, *A* and *E*), suggesting that additional factors might play a role at increasing α-synuclein aggregation.

Previously, it has been reported that BAPTA-AM can inhibit proteases (38–40), which is mediated via blocking intracellular calcium transients required to regulate protease activity (41, 42). This led us to test the effect of mitochondrial proteostasis on α-synuclein pathology. We treated YFP–α-synuclein overexpressing SH-SY5Y cells with CDDO-Me to inhibit Lon protease (43), which has recently been shown to influence aggregate dissolution after heat shock (44), and with UCF-101 to inhibit high temperature requirement protein A2 (HtrA2/Omi) protease (45), which has previously been linked to PD. Our results show that both mitochondrial protease inhibitors significantly increase α-synuclein pathology (Fig. 3*C*), with the effect of HtrA2 protease inhibition on aggregation being higher than the one of Lon protease inhibition.

### Inhibition of mitochondrial proteostasis increases amyloid-β 1-42 pathology

**Figure 4.**
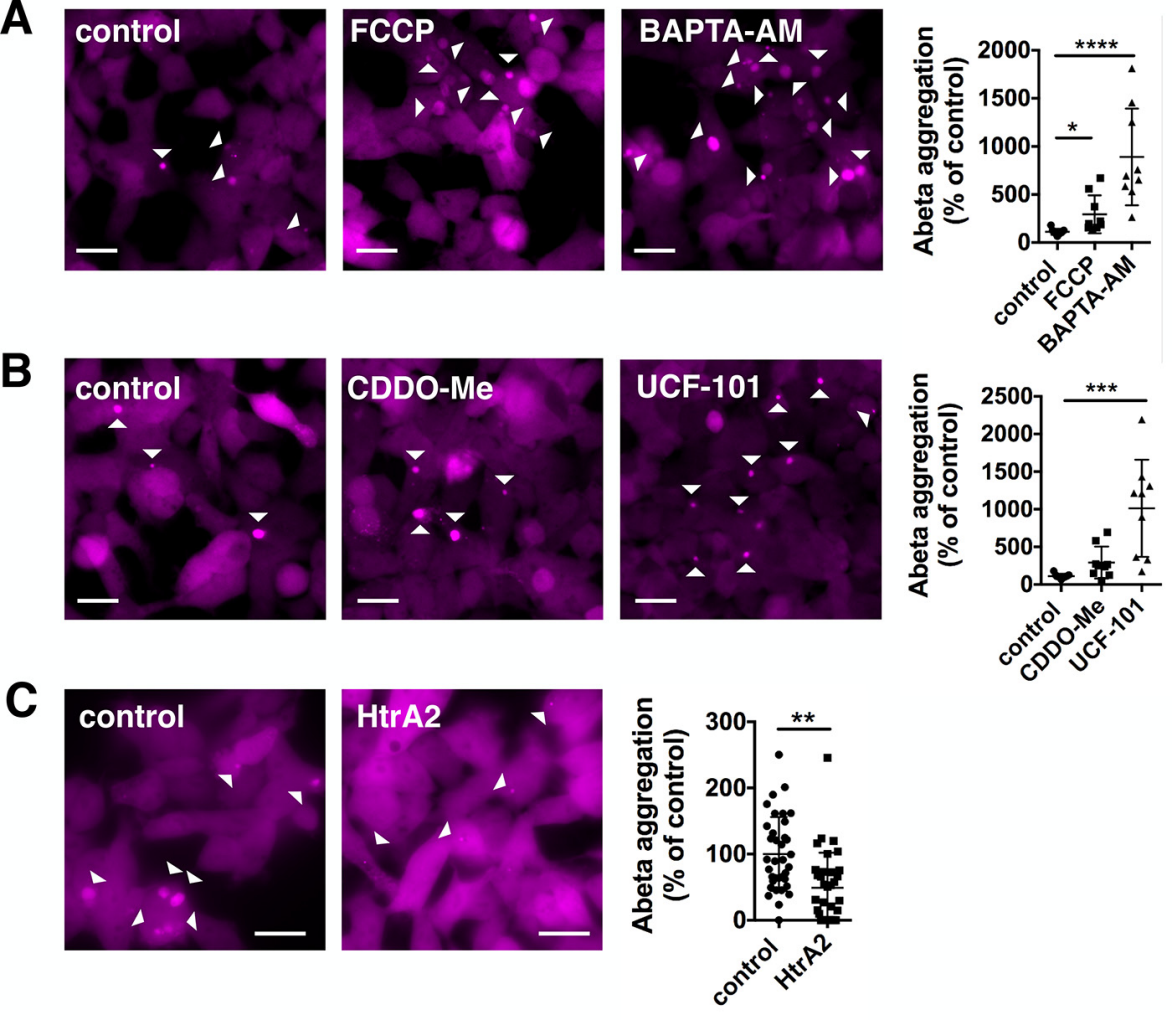
**Mitochondrial proteostasis influences amyloid-β 1-42 pathology.** *A,* Aβ42-mCherry overexpressing HEK cells were treated with DMSO (control), 1 μm FCCP, or 10 μm BAPTA-AM for 24 h. The aggregation of Aβ42 was increased upon treatment with FCCP and BAPTA-AM. Data are presented as mean ± S.D. *, *p* = 0.0298 and ****, *p* < 0.0001 (Kruskal-Wallis test with Dunn's multiple comparison). *n* = 9 for all conditions, with *n* = wells analyzed, three biological repeats. *B,* Aβ42-mCherry cells were treated with DMSO (control), 0.1 μm CDDO-Me, or 20 μm UCF-101 for 24 h. The aggregation of Aβ42 was increased upon treatment with UCF-101. Data are presented as mean ± S.D. ***, *p* = 0.0001 (one-way ANOVA with Dunnett's post hoc correction). *n* = 9 for all conditions, with *n* = wells analyzed, three biological repeats. *C,* Aβ42-mCherry cells were transfected with either uncut pcDNA3 (control) or HtrA2 pcDNA3 and Aβ42-mCherry expression was induced with tetracycline for 3 days. The aggregation of Aβ42 was significantly decreased upon overexpression of HtrA2. Data are presented as mean ± S.D. **, *p* = 0.0089 (two-tailed unpaired *t* test). *n* = 36 and 34 with *n* = images analyzed, four biological repeats. *Scale bars*: 20 μm.

To test if the above-discussed mechanisms also contribute to the aggregation of other proteins involved in neurodegeneration, we investigated the effect of mitochondrial proteostasis on amyloid-β 1-42 (Aβ42) aggregation. We used a stable HEK293 cell line overexpressing Aβ42-mCherry via a tetracycline-inducible expression system, which is described in detail in Ref. 46. After induction of Aβ42-mCherry expression, the cells were treated with FCCP, BAPTA-AM, and the protease inhibitors CDDO-Me and UCF-101. We found that treatment of cells with both FCCP and BAPTA-AM increased the aggregation of Aβ42 (Fig. 4*A*). BAPTA-AM had a more pronounced effect to enhance Aβ42 aggregation compared with FCCP, similar to what had been seen for α-synuclein. Inhibition of the Lon protease did not significantly increase Aβ42 aggregation, however, inhibition of HtrA2 using UCF-101 again increased Aβ42 aggregation (Fig. 4*B*). To test whether increased mitochondrial proteostasis via HtrA2 is indeed able to influence protein aggregation we overexpressed HtrA2 in Aβ42-mCherry HEK cells. After transfection of the cells with HtrA2 and 3 days of induction of Aβ42-mCherry expression we observed a significant reduction of Aβ42 aggregation (Fig. 4*C*).

### In vitro aggregation of amyloid-β 1-42 is influenced by mitochondria and HtrA2

**Figure 5.**
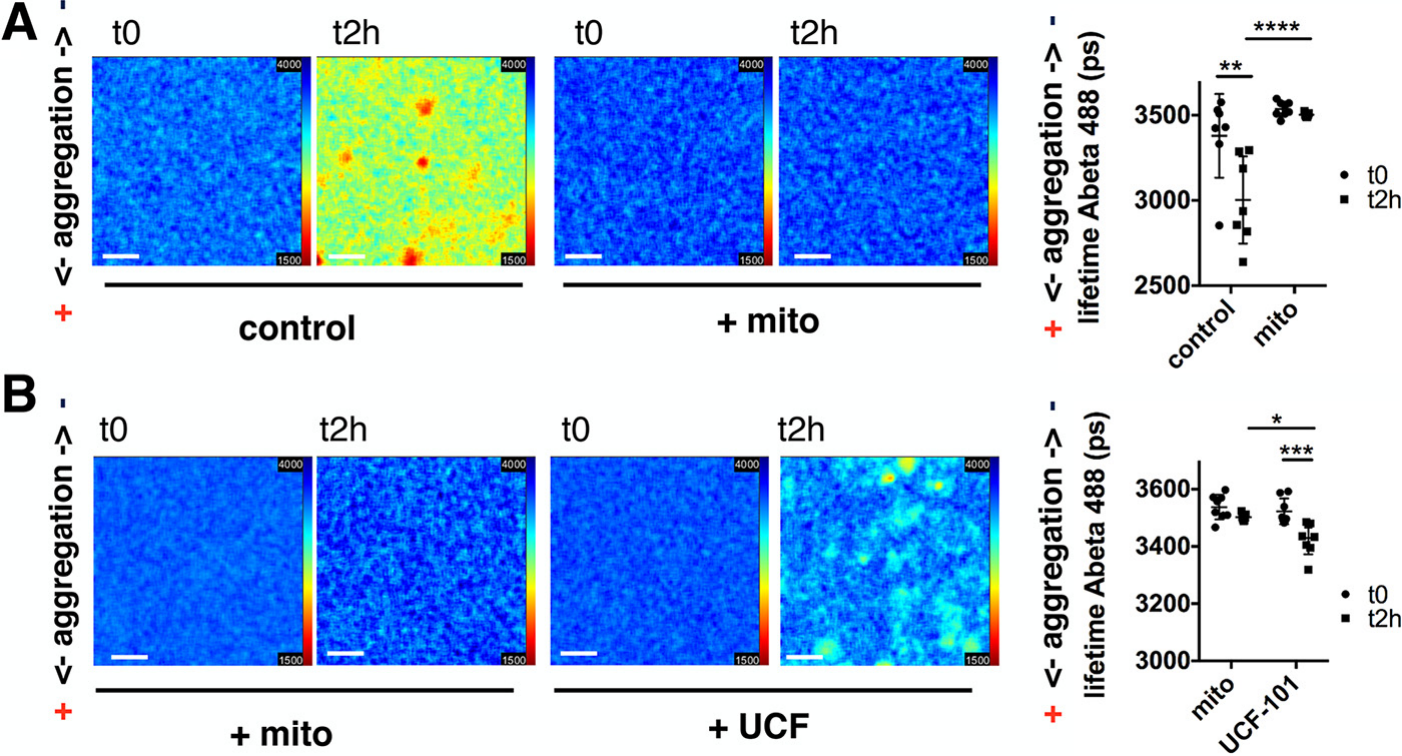
**HtrA2 influences *in vitro* aggregation of Aβ42.** *A,* fluorescence lifetime images of Hilyte™ Fluor 488-labeled Aβ42 at the beginning of the experiment (*t0*) and after 2 h of incubation at room temperature (*t2h*) show a decrease in fluorescence lifetime in control conditions demonstrating protein aggregation. No decrease in Aβ42 Hilyte™ Fluor 488 fluorescence lifetime was seen when isolated mitochondria were present. Data are presented as mean ± S.D. **, *p* = 0.0025 and ****, *p* < 0.0001 (one-way ANOVA with Tukey's post-hoc correction). *n* = 7, 8, 7, and 7 with *n* = wells analyzed, three biological repeats. *Scale bars*: 20 μm. *B,* fluorescence lifetime images of Hilyte™ Fluor 488-labeled Aβ42 at the beginning of the experiment (t0) and after 2 h of incubation at room temperature (*t2h*), showing a decrease in the Aβ42 Hilyte™ Fluor 488 fluorescence lifetime when UCF-101 treated mitochondria were present. Data are presented as mean ± S.D. ***, *p* = 0.0009 and *, *p* = 0.0142 (one-way ANOVA with Tukey's post-hoc correction). *n* = 8, 8, 7, and 8 with *n* = wells analyzed, three biological repeats. *Scale bars*: 20 μm.

To show that mitochondria directly influence protein homeostasis, we chose to investigate Aβ42 aggregation *in vitro* using a fluorescence lifetime aggregation assay. Although α-synuclein aggregation occurs within days (see ThT assays in Fig. 1, *C* and *F*), Aβ42 shows very fast aggregation kinetics (within hours), which permitted us to investigate the effect of isolated brain mitochondria (viable for only several hours). The fluorescence lifetime assay we have used analyses the reduction in fluorescence lifetime of labeled proteins when they start to aggregate and are tightly packed, as previously described in detail (47, 48). We used Aβ42 containing 50% Hylite^TM^ Fluor 488-labeled Aβ42, which was incubated for 2 h at room temperature, after which we measured a reduction of Hylite^TM^ Fluor 488 fluorescence lifetime from 3380 ± 93 to 3003 ± 97 ps (Fig. 5*A*, *control t0* and *t2h*). However, in the presence of isolated rat brain mitochondria, only a small drop in Aβ42 Hylite^TM^ Fluor 488 fluorescence lifetime was detected (Fig. 5*A*, *mito t0* and *t2h*, 3538 ± 15 ps compared with 3502 ± 5 ps). Note, the fluorescence lifetime of Aβ42 Hylite^TM^ Fluor 488 incubated with mitochondria is higher at the beginning of the experiment (t0) than in the control group (Aβ42 + mito 3538 ± 15 ps *versus* Aβ42 control with 3380 ± 93 ps), because in the control Aβ42 starts to aggregate immediately upon preparation, which was not the case in the presence of mitochondria. We next preincubated mitochondria with UCF-101 and showed that the Aβ42 Hylite^TM^ Fluor 488 fluorescence lifetime significantly decreased over the 2 h time interval, demonstrating that Aβ42 aggregation was increased upon inhibition of HtrA2 (Fig. 5*B*, *UCF-101 t0* and *t2h*, 3523 ± 16 *versus* 3429 ± 20 ps).

### Inhibition of mitochondrial protein import enhances α-synuclein and amyloid-β 1-42 pathology

**Figure 6.**
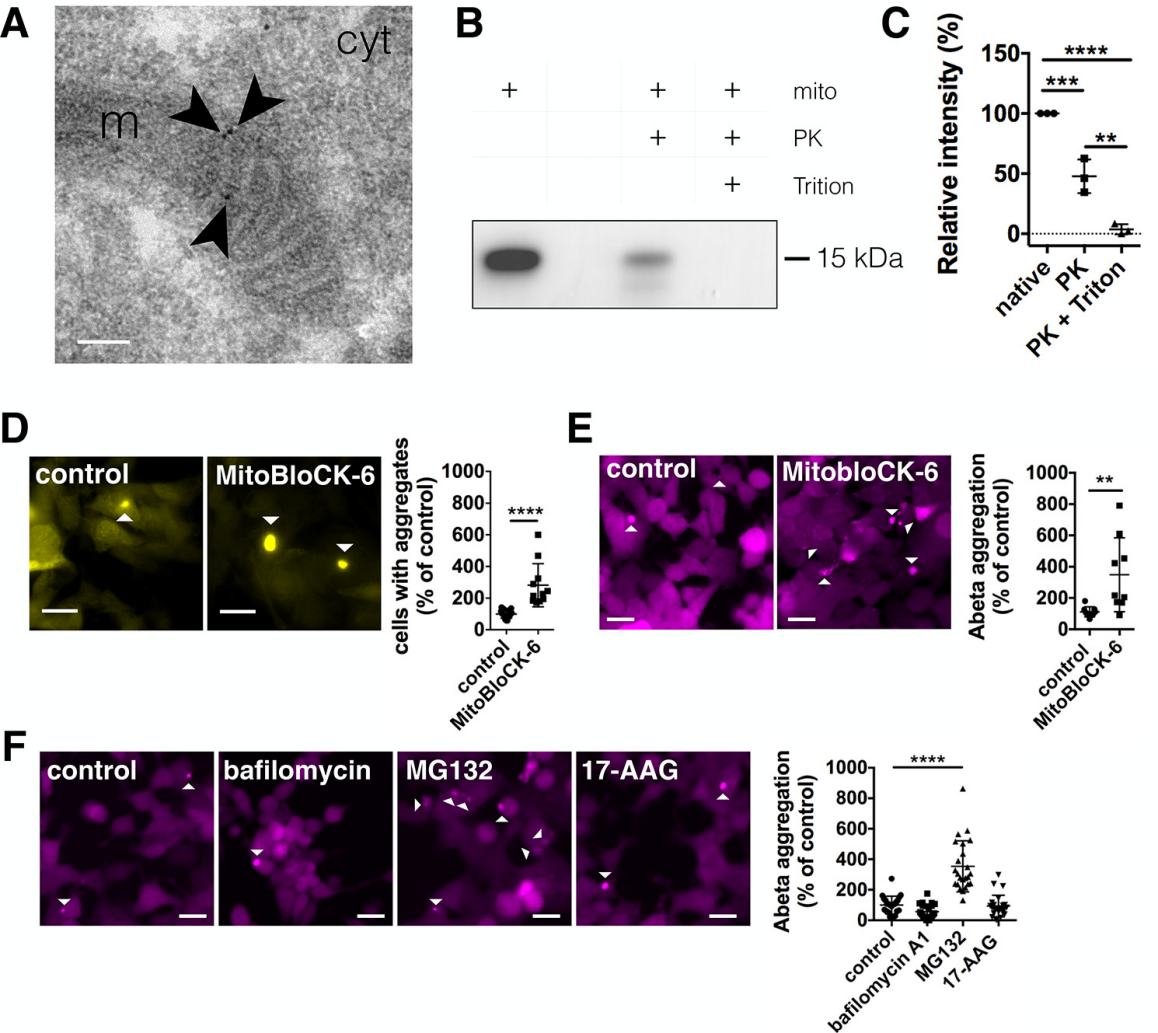
**Inhibition of mitochondrial protein import increases α-synuclein and Aβ42 pathology.** *A,* transmission EM image of immunogold-labeled YFP-α-synuclein in SH-SY5Y cells showing that α-synuclein is contained within mitochondria. *Arrows* indicate individual immunogold labels within mitochondria. *m* = mitochondria, *cyt* = cytoplasm. *Scale bar*: 100 nm. *B,* α-synuclein of isolated mitochondria from adult rat brain in the absence (native) of proteinase K (PK), in the presence of PK, or in the presence of both PK and 0.1% Triton X-100. *C,* relative intensity of α-synuclein bands normalized to native mitochondria. Data are presented as mean ± S.D. ***, *p* = 0.0007; ****, *p* = < 0.0001; **, *p* = 0.0017 (one-way ANOVA with Tukey's post hoc correction). *n* = 3 for all conditions with *n* = biological repeats. *D,* YFP–α-synuclein SH-SY5Y cells were treated with DMSO (control), or 50 μm MitobloCK-6 before and during the incubation with α-synuclein fibrillar seeds (5 h). *Scale bars*: 20 μm. α-Synuclein seeding was significantly increased upon treatment. Data are presented as mean ± S.D. ****, *p* < 0.0001 (two-tailed Mann-Whitney *U* test). *n* = 15, 11 with *n* = regions analyzed, three biological repeats. *E,* Aβ42-mCherry cells were treated with DMSO (control) or 5 μm MitobloCK-6 for 24 h. *Scale bars*: 20 μm. The aggregation of Aβ42 was increased upon treatment with MitobloCK-6. Data are presented as mean ± S.D. **, *p* = 0.0088 (two-tailed unpaired *t* test). *n* = 9 for all conditions, with *n* = wells analyzed, three biological repeats. *F,* Aβ42-mCherry cells were treated with DMSO (control), 10 nm bafilomycin A1, 0.5 μm MG132 or 50 nm 17-AAG for 24 h. *Scale bars*: 20 μm. The aggregation of Aβ42 was significantly increased upon treatment with MG132. Data are presented as mean ± S.D. ****, *p* < 0.0001 (Kruskal-Wallis test with Dunn's multiple comparison). *n* = 23, 24, 24, and 24 with *n* = images analyzed, three biological repeats.

There is recent evidence in the literature that mitochondrial proteases can influence aggregate dissolution and that aggregation-prone proteins are directed to mitochondrial import (44). There is one report showing mitochondrial import of α-synuclein (49), but it is still discussed critically that aggregation-prone proteins, like α-synuclein, are directly imported into mitochondria (50, 51). Thus, to prove that α-synuclein resides within mitochondria, we immunogold-labeled YFP–α-synuclein in SH-SY5Y cells, and found specific staining within mitochondria, which was mainly located at the inner mitochondrial membrane (Fig. 6*A* and Fig. S2). In addition, we isolated mitochondria from WT adult rat brain and probed them for the presence of endogenous α-synuclein after proteinase K (PK) digestion. We see that α-synuclein is still present after PK treatment, indicating that α-synuclein resides within the organelle because PK is not able to degrade proteins protected by organelle membranes. This is further supported by the finding that incubation with 0.1% Triton X-100 during PK treatment, which is capable of solubilizing mitochondrial membranes (52), enables complete α-synuclein degradation (Fig. 6, *B* and *C*).

Because the above results indicated that α-synuclein was localized to mitochondria, we hypothesized that inhibition of mitochondrial protein import might have a similar effect on α-synuclein pathology as the inhibition of proteases. Thus, using MitobloCK-6, a small molecule inhibitor of protein translocation into mitochondria (53), we also observed increased α-synuclein aggregation in YFP–α-synuclein overexpressing SH-SY5Y cells (Fig. 6*D*). Testing MitobloCK-6 on Aβ42-mCherry overexpressing HEK cells again showed increased Aβ42 aggregation (Fig. 6*E*), demonstrating that mitochondrial protein import influences the proteostasis of amyloidogenic proteins.

To test how cytosolic protein homeostasis is influenced by other protein quality control pathways, we used Aβ42-mCherry overexpressing HEK cells and inhibited autophagy using bafilomycin A1, the ubiquitin-proteasome system (UPS) using MG132 and the cytosolic chaperone Hsp90 using 17-AAG. Although bafilomycin A1 and 17-AAG did not increase Aβ42 aggregation, inhibition of the UPS using MG132 increased the aggregation of Aβ42 (Fig. 6*F*), which is in accordance with previous reports (54–56).

## Discussion

We demonstrate here that inhibition of the mitochondrial proteases HtrA2 and Lon, as well as inhibition of the mitochondrial protein import enhances α-synuclein pathology. However, downstream effects of mitochondrial dysfunction, induced without effects on the mitochondrial network, did not recapitulate increased α-synuclein pathology. Inhibition of HtrA2 and mitochondrial protein import further increased Aβ42 pathology, and overexpression of HtrA2 was able to decrease Aβ42 aggregation notably. It was reported recently that mitochondria were able to influence the degradation and protein homeostasis of cytosolic proteins, which has been shown in yeast cells upon heat shock (44). Mitochondria may also play an important role for the degradation of amyloidogenic proteins, because mitochondrial proteostasis seems to be clearly coupled to the pathology of α-synuclein and Aβ42. HtrA2 appears to be of particular interest, because it has previously been linked genetically to PD (57–61) and shows a neuroprotective effect upon overexpression in mice (62, 63).

So far the effect of amyloid proteins on mitochondria has been interpreted only as a secondary pathological hallmark, with α-synuclein as well as Aβ exacerbating mitochondrial dysfunction (50, 51, 64–68). However, amyloidogenic proteins may be deliberately directed to mitochondria, and thereby disrupt overall mitochondrial function if uptake is overloaded. Vice versa, an initial failure in mitochondrial function, *i.e.* by severe complex I inhibition or upon disturbance of mitophagy, can eventually lead to increased levels of α-synuclein, having important implications for sporadic forms of the disease. Our recently published review provides more insight into mitochondrial uptake of α-synuclein and Aβ, on the interaction with mitochondrial translocases as well as background information on mitochondrial proteases (69). In addition to what is already known, our study here shows that mitochondrial proteostasis can influence the aggregation of α-synuclein after seeding. Thus far, it has only been shown that α-synuclein is taken up into mitochondria (49), but not that intra-mitochondrial proteases influence α-synuclein aggregation propensity. Furthermore, our findings refer to α-synuclein seeding. This is especially important, because seeding is understood as a major mechanism during the progression of PD and thus targeting mitochondrial proteostasis in patients may thus be a promising approach to tackle PD. For Aβ, several studies, especially from the laboratory of Elzbieta Glaser (70–72), show that Aβ can be taken up into mitochondria and mitochondrial proteases can influence protein aggregation. Here we present new data on HtrA2 and add further evidence that mitochondrial proteostasis is indeed of physiological and pathophysiological relevance for neurodegenerative diseases.

There still remains the argument that inhibition of mitochondrial proteases just causes unspecific mitochondrial dysfunction, which then *per se* leads to increased α-synuclein aggregation. However, it seems that this effect is not mediated via the known downstream events of mitochondrial dysfunction. Indeed, ATP depletion is not able to increase α-synuclein aggregation, as seen in our study using short-term complex I inhibition via MPP^+^ (where MPP^+^ reduced ATP levels, but did not lead to major mitochondrial fragmentation). Furthermore, a previous study shows that there is no elevated toxicity when ATP levels are reduced independently from mitochondrial respiration using 2-deoxyglucose, which inhibits cytosolic glycolysis (73). Furthermore, increased calcium concentrations, when induced acutely via calcium influx through the plasma membrane using the ionophore ionomycin, did not influence α-synuclein aggregation after seeding. This, in the first instance, seems to stand in contrast to our previous study (24), where we have shown that calcium affects α-synuclein aggregation *in vitro*. However, we saw that mainly the nucleation rate was increased, thus how fast new aggregates are formed, but not the growth rate, *i.e.* how fast aggregates grow from an existing seed. Taken together this implies that calcium may contribute to PD via α-synuclein seed formation, but less to the growth from already formed α-synuclein seeds. Oxidative stress has been discussed as a likely mechanism in PD, because antioxidants are able to reduce dopaminergic neuron death and α-synuclein accumulation after complex I inhibition (73), however, also a general protective impact on mitochondria may play a role.

Although, this does not mean that complex I inhibition, calcium dysregulation, and oxidative stress are not important in disease. Chronic complex I inhibition has clearly been shown to lead to dopaminergic neuron death and α-synuclein accumulation (12–16) and is a major factor implicating mitochondrial dysfunction in sporadic Parkinson's disease. Chronic complex I inhibition can impact mitochondrial fitness and mitochondrial fitness can also be reduced upon high calcium loads as recently demonstrated for dopaminergic neurons of the substantia nigra (74). Taken together, our study shows that mitochondrial proteostasis may be an important factor contributing to the pathology of neurodegenerative diseases, and attacking mitochondrial fitness, rather than downstream events of mitochondrial dysfunction may be crucial in the search for therapeutic strategies.

## Experimental procedures

### Human cell cultures

Human neuroblastoma cells (SH-SY5Y) were obtained from the European Collection of Cell Cultures (ECACC, Sigma-Aldrich, Dorset, United Kingdom) and grown in a 1:1 minimal essential medium (MEM) (Sigma-Aldrich) and nutrient mixture Ham's F-12 (Sigma-Aldrich) supplemented with 15% FBS, 1% nonessential amino acids, 2 mm GlutaMAX, and 1% antibiotic-antimycotic (all Thermo Fisher Scientific, Epsom, United Kingdom). SH-SY5Y cells stably expressing YFP–α-synuclein were obtained by lentiviral transfection using 3rd generation lentiviruses (Addgene constructs: 12251, 12253, and 12259). pMDLg/pRRE, pRSV-Rev, and pMD2.G were a gift from Didier Trono (Addgene plasmid numbers 12251, 12253 and 12259; RRID:Addgene_12251, RRID:Addgene_12253, and RRID:Addgene_12259) (75). Human WT α-synuclein was inserted into EYFP plasmid (pEYFP-N1) using a 5-amino acid linker (sequence: GCACCGGTCGCCACC) between the C terminus of α-synuclein and N-terminal EYFP. α-Synuclein–EYFP was then cloned into the pLJM1 backbone for lentiviral expression (Addgene: 19319). pLJM1-EGFP was a gift from David Sabatini (Addgene plasmid number 19319; RRID:Addgene_19319) (76). For the preformed fibril (PFF) assay, 50,000 cells were plated in MatTek dishes (P35G-1.5-14-C, MatTek Corp., Ashland, MA, USA). For analysis of mitochondrial fragmentation cells were plated at 20,000 cells per well in NuncTM Lab-Tek^TM^ II Chambered Coverglass (8 well, 155409, Thermo Fisher Scientific).

Flp-In^TM^ T-REx^TM^ 293 cell line (Invitrogen), a derivative of HEK293 cells containing a stably integrated FRT site and a TetR repressor, was used to generate stable cell lines expressing either mCherry or Aβ42-mCherry (pcDNA3.3-mCherry, pcDNA3.3-Ab42-mCherry) under the Flp-In^TM^ expression vector as described previously (46, 77). Cells were maintained in DMEM high glucose media (Sigma-Aldrich) supplemented with 10% fetal bovine serum (FBS), 2 mm GlutaMAX, and 1% antibiotic-antimycotic (all Thermo Fisher Scientific). Cells were grown at 37 °C under a 5% CO_2_ atmosphere. Cells were plated at 35,000 cells per well in NUNC 24-well–plates, and construct expression was induced for 3 days using media above with 1 µg/ml of tetracycline (Sigma-Aldrich) added. All cell lines were tested for mycoplasma contamination using the MycoAlert^TM^ PLUS mycoplasma detection kit (Lonza, Walkersville, MD). For transient transfection of HtrA2 electroporation with the NEON transfection system was used (settings: 1050 V, 30 ms, 2 pulses; Thermo Fisher Scientific). pcDNA3-HtrA2-FLAG was a gift from L. Miguel Martins (Addgene plasmid number 15938; RRID:Addgene_15938) (78).

Cells were imaged on a widefield microscope with IX83 frame (Olympus, Tokyo, Japan), HPLS343 plasma light source (Thorlabs, Newton, NJ, USA), and Clara interline CCD camera (Andor, Belfast, United Kingdom), controlled by Micromanager (79). Respective filter cubes for YFP (excitation 500 nm, dichroic mirror 515 nm, emission 535 nm), RFP (excitation 560 nm, dichroic mirror 585 nm, emission 630 nm), and 4″,6-diamidino-2-phenylindole (excitation 350 nm, dichroic mirror 400 nm, emission 460 nm) were used. Images for YFP–α-synuclein aggregation and 4′,6-diamidino-2-phenylindole were taken with an Olympus Plan Apo U ×60/1.42 oil objective lens. Imaging was done randomly by automated acquisition of a grid of 7 × 7 images per area. Aggregates were identified by their fibrillar nature, cell nuclei were counted using FIJI (80). For Aβ42-mCherry aggregation images were taken with an Olympus LUCPlanFLN ×20/0.45 air objective lens. Aggregates were identified using the Thresholder plugin in ICY (81). The cell surface area was evaluated using the HK-Means plugin for ICY (82).

### α*-Synuclein fibrils*

Human WT (WT) α-synuclein was expressed in *Escherichia coli* One Shot^®^ BL21 STAR™ (DE3) (Invitrogen, Thermo Fisher Scientific) cells using plasmid pT7-7 and purified using ion-exchange on a HiPrep Q FF 16/10 anion exchange column (GE Healthcare, Uppsala, Sweden) (83). α-Synuclein was then further purified on a HiPrep Phenyl FF 16/10 (High Sub) hydrophobic interaction column (GE Healthcare) (84). Purification was performed on an ÄKTA Pure (GE Healthcare). Monomeric protein was dialyzed against 20 mm phosphate buffer, pH 7.2, lyophilized in a LyoQuest 85 freeze-dryer (Telstar, Spain), and stored at −80 °C.

α-Synuclein fibrils were produced by diluting α-synuclein monomer solution to a concentration of 150 μm in 20 mm phosphate buffer, pH 7.2. Samples were incubated at 37 °C for 5 days in 0.5-ml Protein Lobind tubes (Eppendorf, Hamburg, Germany) under continuous rotation at maximum speed (UVP HB-1000 Hybridizer, Fisher Scientific). Fibrils were diluted 1:1 with 20 mm phosphate buffer, pH 7.2, to a final volume of 200 μl and sonicated (Digital Sonifier^®^ SLPe, model 4C15, Branson, Danbury, MA, USA) with six 10-s pulses at 70% amplitude and 10-s pause after each sonication pulse. Sonicated fibrils were aliquoted, exposed to UV light for 30 min, and frozen immediately after at −80 °C.

α-Synuclein fibrils were imaged by atomic force microscopy (BioScope Catalyst microscope, Bruker AXS GmbH, Fitchburg, USA). Fibrils at an equivalent monomer concentration of 5 μm were deposited for 30 min on High Performance coverglass (PN 474030-9020-000, Carl Zeiss Ltd.), cleaned for 30 min with 1 m KOH (Fluka, Bucharest, Romania), and coated for 30 min with 0.01% poly-l-lysine beforehand (P4707, Sigma). Samples were rinsed 5 times with deionized water and dried under nitrogen flow. Atomic force microscopy data were acquired using PeakForce Quantitative Nanomechanical Property mapping mode with ScanAsyst-Fluid+ probes (BioScope Resolve, Bruker AXS GmbH). Images were flattened and exported using NanoScope Analysis software, version 1.8.

### Preformed fibril (PFF) assay

For the induction of α-synuclein seeding, YFP–α-synuclein overexpressing SH-SY5Y cells were incubated with sonicated preformed α-synuclein fibrils as described by Luk *et al*. (25). Briefly, cells plated in MatTek dishes were washed with Neurobasal medium and subsequently changed to 500 μl of Neurobasal medium supplemented with 2% B27 and 0.5 mm GlutaMAX (all Thermo Fisher Scientific). Cells were preincubated for 1 h, either using DMSO for control or the respective treatment (see cell treatments below). 8 μl of PFFs were diluted with 32 μl of Hanks' balanced salt solution (Hanks' balanced salt solution minus calcium and magnesium, no phenol red, 14175-053, Thermo Fisher Scientific) and mixed briefly 5 times. Fibrils were added to the bottom of the BioPORTER tube (BioPORTER^®^ Protein Delivery agent, BP502424, Gelantis, San Diego, CA, USA), mixed 5 times, and incubated for 5 min at room temperature, then vortexed for 5 s at 600 rpm (Stuart^TM^ Scientific SA8 vortex mixer, Sigma-Aldrich). 460 μl of OptiMEM (Thermo Fisher Scientific) was added to the BioPORTER tube plus the respective treatments and mixed 5 times. The PFF mixture was added dropwise to the cells, settled, and then incubated for 4 h at 37 °C and 5% CO_2_. The final monomer equivalent concentration of preformed fibrils was 600 nm.

After 4 h, cells were washed twice with 1 ml of Neurobasal medium and changed subsequently to 2 ml of retinoic acid medium made of 1:1 MEM (Sigma-Aldrich) and nutrient mixture Ham's F-12 (Sigma-Aldrich) supplemented with 5% FBS, 1% nonessential amino acids, 2 mm GlutaMAX, 1% antibiotic-antimycotic (all Thermo Fisher Scientific), and 1 μm retinoic acid (Sigma-Aldrich) plus treatments if indicated and incubated for another 3 days to allow aggregate formation. Cells were fixed for 10 min using 4% formaldehyde in PBS supplemented with 4% sucrose, 5 mm MgCl_2_, and 10 mm EGTA, pH 7.4 (85), and stained with Hoechst 33342 (Molecular Probes, Thermo Fisher Scientific) 1:2000 in PBS for 30 min.

### Cell treatment

Chemicals used for the treatment of cells were prepared as followed, with final dilutions made with the respective cell culture medium. FCCP **(**Abcam, Cambridge, UK), 1 mm in DMSO, MPP^+^ **(**Sigma-Aldrich), 10 mm in water, ionomycin (ab120370, Abcam) 10 mm, and 1 mm in DMSO, 2-deoxyglucose (Sigma-Aldrich) 0.5 **m** in water, menadione (Sigma-Aldrich), 1.5 mm in DMSO, BAPTA-AM (ab120503, Abcam), 2.5 mm in DMSO, BAPTA (ab144924, Abcam), 1 mm in water, CDDO-Me (Sigma-Aldrich), 1 mm in DMSO, UCF-101 (Sigma-Aldrich), 10 mm in DMSO, MitobloCK-6 (Focus Biomolecules), 5 mm in DMSO, bafilomycin A1 (Calbiochem, San Diego, CA, USA), 100 and 10 μm in DMSO, 17-AAG (ab141433, Abcam), 5 mm and 50 μm in DMSO and MG132 (Sigma-Aldrich), and 10 and 1 mm in DMSO.

### Immunofluorescence

Cells were fixed as described above, blocking and permeabilization were performed using 5% donkey serum in 0.05% Tween-20 in PBS (PBS) for 1 h. Primary antibodies were incubated overnight at 4 °C, followed by 5 washes with PBS. Secondary antibodies were incubated for 1 h at room temperature, followed by 5 washes with PBS. As primary antibodies anti-ubiquitin antibody, clone Apu2 (05-1307, 1:200, Millipore, Watford, United Kingdom), anti-ubiquitin-binding protein p62, clone 2C11 (SQSTM1, 1:200, Abnova, Taipei, Taiwan), and anti-FLAG^®^ M2 antibody (F1804, 1:200, Sigma-Aldrich) were used. As secondary antibodies anti-rabbit and anti-mouse Alexa Fluor^®^ 647, and anti-mouse Alexa Fluor^®^ 568 (A-21245, A-21236 and A-11031 from Life Technologies) were used. Samples were kept in PBS containing 5 mm sodium azide (Sigma-Aldrich).

### Structured illumination microscopy (SIM)

Structured illumination images were collected on a custom-built structured illumination microscopy (SIM) setup, which has been described in detail (86). A ×60/1.2 NA water immersion lens (UPLSAPO 60XW, Olympus) focused the structured illumination pattern onto the sample was used. This lens also captured the samples' fluorescence emission light before imaging onto a sCMOS camera (C11440, Hamamatsu). Laser excitation wavelengths used were 488 (iBEAM-SMART-488, Toptica), 561 (OBIS 561, Coherent), and 640 nm (MLD 640, Cobolt). Respective emission filters were BA 510-550 (Olympus), BrightLine FF01-600/37, and BrightLine FF01-676/29 (Semrock, New York, USA). Imaging was done on fixed or live cells, as indicated. Images were acquired using custom SIM software (HCImage, Mamamatsu Corporation, Sewickley, NJ, USA). Nine raw images were collected at each plane and each color. FairSIM plugin in FIJI was used to reconstruct images (87).

### FLIM measurements of cytosolic calcium, H_2_O_2_, and ATP

Fluorescence lifetime microscopy (FLIM) was carried out on a custom-built time-correlated single photon counting system using a super-continuum laser (SC450, Fianium) with a pulse repetition rate of 40 MHz, a confocal scanning unit (FluoView 300, Olympus) coupled with an inverted microscope frame (IX70, Olympus), and a time-correlated single-photon counting system (Becker & Hickl GmbH) as described in detail (88). The excitation wavelength was selected using an acousto-optic tunable filter (AOTFnC-400.650, Quanta Tech) and respective excitation filters (to improve the wavelength selection) and emission fluorescence were imaged through respective emission filters. The data acquisition time was 200 s for each FLIM image (10 cycles, 20 s/cycle). The photon detection rate was kept below 2% of the laser repetition rate to avoid photon pile-up.

For cytosolic calcium measurements SH-SY5Y cells were incubated with Oregon Green^TM^ 488 BAPTA-1, AM (Thermo Fisher Scientific) for 45 min at 1 μm concentration. Excitation was set to 475 nm, excitation filter BrightLine FF01-474/27 (Semrock), and emission filter BrightLine FF01-525/39 (Semrock) were used. For measurements of H_2_O_2_ and ATP, SH-SY5Y cells were transiently transfected with the respective sensor using electroporation with the NEON transfection system (settings: 1100 V, 50 ms, 1 pulse; Thermo Fisher Scientific). HyPer, a genetically encoded sensor consisting of circularly permuted yellow fluorescent protein inserted into the regulatory domain of the prokaryotic H_2_O_2_-sensing protein, OxyR (35), was used to measure cytosolic hydrogen peroxide. Excitation was set to 470 nm, the same excitation and emission filters as for Oregon Green^TM^ 488 BAPTA-1 were used. Ateam1.03, a FRET-based indicator for ATP composed of the ε subunit of the bacterial F_o_F_1_-ATP synthase sandwiched by CFP and YFP (33, 34) was used to measure cytosolic ATP levels. Excitation was set to 435 nm, excitation filter BrightLine FF01-434/17 (Semrock), and emission filter BrightLine FF01-470/28 (Semrock) were used. ATeam1.03-nD/nA/pcDNA3 was a gift from Takeharu Nagai (Addgene plasmid number 51958; RRID:Addgene_51958). For ATP measurements, cells were subjected to media containing 10 mm 2-deoxyglucose to inhibit glycolysis. The fluorescence lifetime was analyzed by the FLIMfit software tool developed at Imperial College London (89, 90).

### ThT assay

The aggregation of α-synuclein *in vitro* was measured by ThT assay. Briefly, 50 μl of 100 μm α-synuclein with 10 μm fresh ThT added was incubated for 7 days with 1% DMSO as a control, 10 μm FCCP, 10 μm BAPTA-AM, or 10 μm BAPTA. Assays were performed in NUNC™ black 384-well plates with optical flat bottoms (142761, Thermo Fisher Scientific), which were sealed with an Ampliseal transparent microplate sealer (Greiner Bio-One GmbH). Plates were incubated including orbital shaking at 300 rpm for 5 min before each read every hour at 37 °C for 170 cycles. The readings of ThT fluorescence intensity were taken using excitation at 440 nm and emission at 480 nm, collected from the bottom with 20 flashes per well and a gain setting of 1300 (FLUOstar Omega, BMG Labtec GmbH, Ortenberg, Germany). Experiments were repeated three times with four replicates for each condition.

### Mitochondrial fragmentation

To label mitochondria, SH-SY5Y cells were incubated overnight with 1:1000 CellLight^TM^ Mitochondria-RFP (Thermo Fisher Scientific) and imaged with a widefield microscope (as described under the section for cell culture). Images were taken randomly by automated imaging of a grid and images from 3 biological repeats were -analyzed. The mitochondrial length was evaluated using the NIEL Mito algorithm (91, 92).

### Animals

Adult female Sprague-Dawley rats were supplied by Charles River UK Ltd., Scientific, Breeding and Supplying Establishment, registered under Animals (Scientific Procedures) Act 1986, and AAALAC International accredited. All animal work conformed to guidelines of animal husbandry as provided by the UK Home Office. Animals were sacrificed under schedule 1; procedures that do not require specific Home Office approval. Animal work was approved by the NACWO and University of Cambridge Ethics Board.

### Mitochondrial isolation and Western blotting analysis

Mitochondria were isolated from adult rat brains by differential centrifugation using the mitochondria isolation kit for tissue (ab110168, Abcam). Western blotting for α-synuclein was performed using 4–12% BisTris gels (Life Technologies), the protein was transferred onto 0.45-μm Millipore PVDF membranes (Fisher Scientific, Loughborough, UK) and subsequently fixed using 4% formaldehyde + 0.1% glutaraldehyde in PBS (both Sigma-Aldrich) (93). As primary antibody α-synuclein (D37A6) XP® rabbit mAb was used (1:1000 dilution, number 4179, CST, Leiden, Netherlands). An enhanced chemiluminescence (ECL)-horseradish peroxidase–conjugated secondary antibody (NA934V, 1:1000 dilution, GE Healthcare, Uppsala, Sweden) and SuperSignal West Femto Chemiluminescent Substrate (Thermo Fisher Scientific) were used to probe the membrane, which was exposed using a G:BOX (Syngene, Cambridge, UK). Western blots were analyzed in FIJI (80).

### Transmission EM

SH-SY5Y cells and SH-SY5Y cells overexpressing YFP–α-synuclein were cultured in 6-well–plates (Greiner Bio-One GmbH) at 350,000 per well. After reaching confluence, cells were washed with 0.9% NaCl (Sigma-Aldrich) twice and incubated with 8% formaldehyde in 0.05 m sodium cacodylate buffer (paraformaldehyde from Merck, Darmstadt, Germany), pH 7.4, for 2 h at 4 °C. Cells were scraped from 6 wells and centrifuged for 10 min at 3,500 × *g*. Cells were washed 5 times in 0.05 m sodium cacodylate buffer, 3 times in deionized water, and incubated with 2% uranyl acetate in 0.05 maleate buffer, pH 5.2 (both BDH Chemicals Ltd., Dorset, UK), overnight at 4 °C. Cells were washed again and dehydrated at increasing ethanol concentrations (1 × 50% EtOH, 3 × 70% EtOH, 3 × 95% EtOH, 3 × 100% EtOH, 3 × 100% dry EtOH; 5 min in each, Sigma-Aldrich). Cells were resuspended in LRW resin (LR White Resin, London Resin (Hard), Agar Scientific, Stansted, UK) mixed 50/50 with dry 100% EtOH, and incubated overnight at room temperature. The following day, cells were spun down, and resuspended in pure LRW for 2 days, where LRW was exchanged twice. Cells were centrifuged at 13,000 × *g* to form a firm pellet, which was transferred to size 2 gelatin embedding capsules (TAAB, Aldermaston, UK) containing LRW resin. Gelatin capsules were covered with a glass coverslip to exclude any air and the resin was cured at 60 °C for 2 days. Gelatin capsules were removed and ultrathin sections were cut using a Leica Ultracut E Ultramicrotome (Leica, Wetzlar, Germany) and placed on 400-mesh nickel/formvar film grids (EM Resolutions). Sections were stained with anti-GFP antibody (ab6556, Abcam) in blocking solution (2% BSA (BBITM solutions, Crumlin, UK) in 10 mm Tris (Sigma-Aldrich) buffer, pH 7.4, containing 0.001% Triton X-100 (Calbiochem, San Diego, CA, USA) and 0.001% Tween 20 (Sigma-Aldrich) at 1:100 overnight. After washing, sections were incubated with goat anti-rabbit 10-nm gold secondary antibody (BBITM solutions) in blocking solution at 1:200 for 1 h. Sections were washed with washing buffer (same as above omitting BSA), deionized water, and left to dry overnight. Post-staining included 2% uranyl acetate in 50% methanol for 30 s, followed by washing with 50% methanol and 30-s staining in Reynold's lead citrate (lead nitrate from BDH Biochemicals Ltd.; trisodium citrate from Sigma-Aldrich). Grids were rinsed thoroughly with deionized water and dried before imaging. Grids were imaged on an FEI Tecnai G2 electron microscope (Thermo Fisher Scientific) run at 200 keV using a 20 μm objective aperture, images were taken using an AMT V600 camera (AMT, Woburn, MA, USA).

### In vitro measurements of Aβ42 aggregation

Synthetic Aβ42 and Aβ42 Hilyte™ Fluor 488 (both from Anaspec, Seraing, Belgium) were prepared as previously described (94). Briefly, lyophilized Aβ42 (1 mg) was dissolved in ice-cold TFA (200 ml), sonicated at 0 °C for 60 s and then lyophilized overnight. Ice-cold 1,1,1,3,3,3-hexafluro-2-propanol (1 ml) was added, sonicated at 0 °C for 60 s, and aliquoted as 20-μl units. The samples were lyophilized overnight and were stored at −80 °C until use. Lyophilized Aβ42 Hilyte™ Fluor 488 peptide (0.1 mg) was dissolved in 1% NH_4_OH (200 μl) and sonicated for 60 s at 0 °C. The sample was aliquoted into 5-μl units, snap-frozen in liquid nitrogen, and stored at −80 °C. Immediately before the experiment unlabeled Aβ42 was prepared by adding first DMSO (5% of total solvent volume), then sodium phosphate buffer (sodium phosphate buffer, 50 mm, pH 7.4) to reach a concentration of 20 μm. The solution was sonicated at 0 °C for 3 min and centrifuged at 13,400 rpm at 0 °C for 30 min. Then the sample was further diluted to 5 μm concentration with sodium phosphate buffer. Also the labeled Aβ42 Hilyte™ Fluor 488 was brought to 5 μm concentration in sodium phosphate buffer and both were mixed in 1:1 ratio. Samples were prepared on ice adding Aβ42, 1 mg/ml of purified mitochondria (preparation see above), and 20 μm UCF-101. Mitochondria isolation buffer and DMSO were added in control samples. 12-μl Volumes were pipetted in silicon gaskets (Thermo Fisher Scientific, P24742) on a coverslip and measured at room temperature. FLIM were carried out on a custom-built time-correlated single photon counting system as described above (see FLIM measurements of cytosolic calcium, H_2_O_2_, and ATP).

### Statistics

Statistical analysis was performed using GraphPad Prism 6.07 (GraphPad Software, Inc., La Jolla, CA, USA). Values are given as mean ± S.D. unless otherwise stated. Normal distribution was tested using a Shapiro-Wilk test. Two-tailed unpaired *t* test was used upon normal distribution, two-tailed Mann-Whitney *U* test was used when no normal distribution was given. For multiple comparisons either one-way ANOVA with Dunnett's post hoc correction upon normal distribution or Kruskal-Wallis test with Dunn's multiple comparison when no normal distribution was given were performed. Significance was considered at *p* < 0.05.

## Data availability

All relevant data are available from the corresponding authors.

## Supplementary Material

Supporting Information
