# Supplementary material for: Intramitochondrial proteostasis is directly coupled to α-synuclein and amyloid β1-42 pathologies
Source: J Biol Chem. 2020 May 8;295(30):10138–52. doi: 10.1074/jbc.RA119.011650 (PMC7383368; doi:10.1074/jbc.RA119.011650)
Supplement: Supporting Information [file supp_295_30_10138__index.html]

Intramitochondrial proteostasis is directly coupled to α-synuclein and amyloid β 1-42 pathologies — Intramitochondrial proteostasisis and α-syn/Aβ42 pathologies — Intramitochondrial proteostasis is directly coupled to α-synuclein and amyloid β1-42 pathologies — Intramitochondrial proteostasisis and α-Syn/Aβ42 pathologies — Supporting Information 

# Intramitochondrial proteostasis is directly coupled to α-synuclein and amyloid β1-42 pathologies

## Supporting Information

- Supporting Information (to be published online) - Supplemental Figures with Legend
