# Supplementary material for: Intramitochondrial proteostasis is directly coupled to α-synuclein and amyloid β1-42 pathologies
Source: J Biol Chem. 2020 May 8;295(30):10138–52. doi: 10.1074/jbc.RA119.011650 (PMC7383368; doi:10.1074/jbc.RA119.011650)
Supplement: Supporting Information [file supp_RA119.011650_156744_2_supp_525923_q9yn69.docx]

**Supplemental Figures**

**Intramitochondrial proteostasis is directly coupled to α-synuclein**

**and amyloid β 1-42 pathologies**

Janin Lautenschläger^1*^, Sara Wagner-Valladolid^1^, Amberley D. Stephens^1^, Ana Fernández-Villegas^1^, Colin Hockings^1^, Ajay Mishra^1^, James D. Manton^2^, Marcus J. Fantham^3^, Meng Lu^1^, Eric J. Rees^2^, Clemens F. Kaminski^3^, Gabriele S. Kaminski Schierle^1*^

^1^ Molecular Neuroscience Group, Department of Chemical Engineering and Biotechnology, University of Cambridge, West Cambridge Site, Philippa Fawcett Drive, Cambridge, CB3 0AS, UK; ^2^ Quantitative Imaging Group, Department of Chemical Engineering and Biotechnology, University of Cambridge, West Cambridge Site, Philippa Fawcett Drive, Cambridge, CB3 0AS, UK; ^3^ Laser Analytics Group, Department of Chemical Engineering and Biotechnology, University of Cambridge, West Cambridge Site, Philippa Fawcett Drive, Cambridge, CB3 0AS, UK

**Supplementary Figure 1**

**Supplementary Fig. 1. Incubation of SH-SY5Y cells overexpressing YFP-alpha-synuclein with alpha-synuclein seeds leads to YFP-alpha-synuclein fibril formation.**

(A) Schematic overview of the cellular alpha-synuclein aggregation/seeding assay.

(B) Fibrillar seeds generated from recombinant human wild-type alpha-synuclein shown by atomic force microscopy before (upper panel) and after sonication (lower panel). Scale bars: 1 µm.

(C) Structured illumination microscopy (SIM) images of SH-SY5Y cells overexpressing YFP-tagged alpha-synuclein in the absence (left) and upon incubation with alpha-synuclein fibrillar seeds (right). Scale bars: 10 µm.

(D and E) Co-staining of YFP-alpha-synuclein fibrillar aggregates with ubiquitin and ubiquitin-binding protein p62. Scale bars: 10 µm.

**Supplementary Figure 2**

**
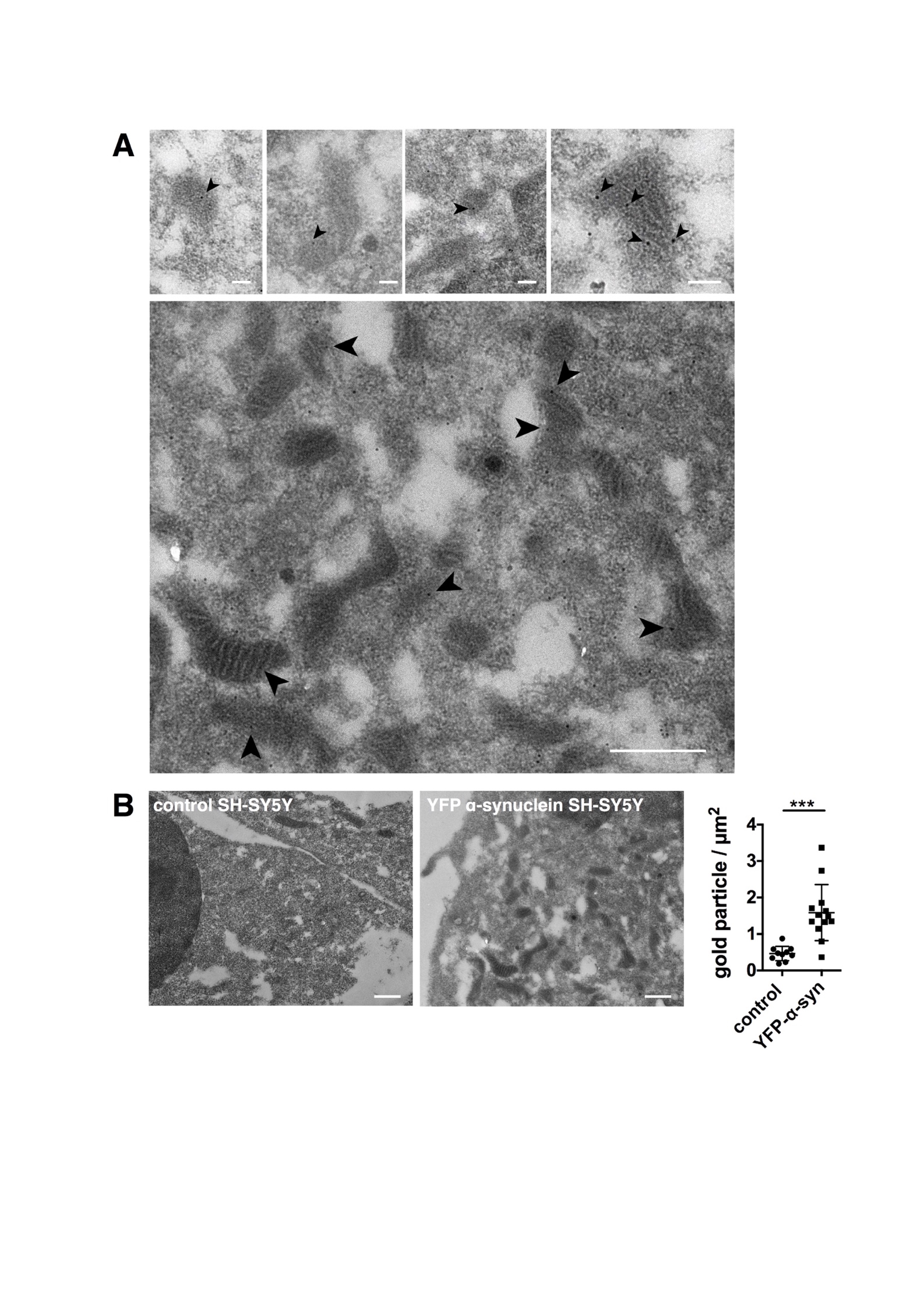
**

**Supplementary Fig. 2. TEM of immunogold labelled YFP-alpha-synuclein, supplementary to Fig. 6A.**

(A) Representative images of transmission electron microscopy (TEM) from SH-SY5Y cells overexpressing YFP-alpha-synuclein showing that alpha-synuclein is contained within mitochondria. Arrows indicate individual immunogold labelling of YFP-alpha-synuclein within mitochondria. Scale bars: 100 nm small images, 500 nm large overview image.

(B) TEM images and quantification of anti-GFP staining in control SH-SY5Y cells and SH-SY5Y cells overexpressing YFP-alpha-synuclein. Quantification of gold particles / µm2 shows that the staining is enriched in YFP-alpha-synuclein overexpressing cells and not due to unspecific background. Data are presented as mean ± SD. ***p = 0.0002 (two-tailed unpaired t-test). N = 10, 13 with n = images analyzed. Scale bars: 500 nm.
